# The effect of *Brachiaria brizantha* cultivars on host-parasite-environment interactions in sheep naturally infected with gastrointestinal nematodes

**DOI:** 10.1371/journal.pone.0238228

**Published:** 2020-08-28

**Authors:** Francisca Fernanda da Silva Roberto, Gelson dos Santos Difante, Lilian Giotto Zaros, Joelma da Silva Souza, Antonio Leandro Chaves Gurgel, Pablo Ramon Costa, Henrique Rocha de Medeiros, Carlikelly Gleicy da Silva, Fernando de Almeida Borges, Neila Lidiany Ribeiro

**Affiliations:** 1 Department of Animal Science, Federal University of Paraíba, Areia, Paraíba, Brazil; 2 Department of Animal Science, Federal University of Mato Grosso do Sul, Campo Grande, Mato Grosso do Sul, Brazil; 3 Department of Animal Science, Federal University of Rio Grande do Norte, Natal, Rio Grande do Norte, Brazil; 4 Department of Animal Science, Federal University of Minas Gerais, Belo Horizonte, Minas Gerais, Brazil; 5 Department of the National Semi-Arid Institute, Campina Grande, Paraíba, Brazil; University of California Riverside, UNITED STATES

## Abstract

The objective was to evaluate the effect of different cultivars of *Brachiaria brizantha* on the dynamics and concentration of the larval stages of gastrointestinal nematodes in the soil and forage strata, as well as their effects on the performance of naturally infected lambs. Overall, 48 90-day-old lambs with an initial weight of 19.04 ± 0.96 kg were observed. Moreover, a randomised block factorial design with four cultivars of *Brachiaria brizantha* (Marandu, Xaraés, Piatã and Paiaguás grasses) under intermittent stocking (with a pre-grazing canopy height of 40 cm and post-grazing canopy height of 20 cm) for two grazing cycles was used. The following variables were analysed: faecal egg counting, faecal culture, mean corpuscular volume, FAMACHA^©^ score, weight and body condition score, the recovery of larvae from pasture and soil samples, nutritional value and the production and structural components of forage. Lambs grazing Marandu grass demonstrated the highest level of nematode infection (*P* < 0.05). However, the nutritional value did not differ between cultivars. Marandu grass had the highest pasture density (*P* < 0.05), while Paiaguás grass had the highest percentage of dead material (*P* < 0.05). The various genera of gastrointestinal nematodes found in the faecal cultures, regardless of the cultivars, include *Haemonchus* (92.01%), *Trichostrongylus* (4.55%), *Strongyloides* (3.06%) and *Oesophagostomum* (0.37%). Lambs grazing Xaraés grass had the lowest body weight (*P* < 0.05). Furthermore, larvae concentrations were highest in Marandu and Paiaguás pastures; infective *Haemonchus* and *Trichostrongylus* larvae were recovered from pasture and soil samples. The different cultivars of *Brachiaria brizantha* produce diverse and relevant microclimatic conditions to contaminate soil, pastures and animals. Animal performance was not compromised despite the Marandu and Paiaguás cultivars having the highest levels of contamination and infection. Based on parasitological aspects, the *Brachiaria brizantha* cultivars Xaraés and Piatã are recommended for grass-based sheep production systems over the other cultivars since they contribute to the reduction of larval contamination and infection.

## Introduction

Infections due to gastrointestinal nematodes (GINs) are a serious global health issue for domestic ruminants and lead to severe economic losses for farmers [1]. Anthelmintic drugs are the most common treatment for GIN infections; however, improper administration and overadministration of these drugs contribute to the development of anthelmintic resistance and result in the deposition of residues in the environment and animal source foods [2].

Since the percentage of parasites is highest in pastures (soil and plant) [3], the microclimate of pastures is indispensable to the development and survival of infective larvae [3, 4]. Methods that prevent or reduce the contact between infective parasites and their hosts should thus be used in livestock systems [5].

Choosing the right forage cultivar and implementing proper grazing management are therefore crucial to sheep farming. In addition to contributing to animal growth, the distinctive morphological characteristics of each cultivar interact with climate conditions (rainfall, temperature, humidity and solar radiation), providing a microclimate suitable for the development of parasites. This microclimate also influences population dynamics and the vertical migration of larvae in the forage strata and even the soil [6].

Cultivars of *Brachiaria brizantha* (Marandu, Xaraés, Piatã and Paiaguás) are among the most used forage plants in livestock systems, as these grasses are flexible and well adapted to soil fertility restrictions and management systems. However, the morphological characteristics of these grasses vary between cultivars and can thus provide a more favourable or unfavourable microclimate for the survival of helminths in the environment.

For example, the Marandu cultivar is different from other cultivars of *Brachiaria brizantha* due to its robustness and phenotypic plasticity at the target canopy height. Moreover, its stems are initially prostrate with intense tillering, and trichomes are located at the apex of the internodes. Marandu grass also has hairy sheaths and wide, long leaf blades with pubescence beneath [7].

The Xaraés cultivar has a caespitose growth habit and early basal tillering. Its stems are green, little branched and 6 mm wide, whereas its leaf blades can be up to 64 cm long and 3 cm wide and shortly pubescent adaxially [8]. The Piatã cultivar is a medium-sized plant with green stems 4 mm wide and aerial tillering. Its leaf blades can be up to 45 cm long and 1.8 cm wide and are not pubescent. Nevertheless, Piatã grass has rough upper leaf surfaces [9]. The Paiaguás cultivar is a medium-sized plant with narrow leaves and thin stems, neither of which are pubescent; aerial tillering and early flowering have been observed. Paiaguás grass has a high potential for animal production in the dry season, a high percentage of leaves and great nutritional value [10].

This study tested whether cultivars of *Brachiaria brizantha*, which have distinct structural characteristics, influence the larval stages of GINs in the soil, plant and host. The objective was thus to evaluate the effect of different cultivars of *Brachiaria brizantha* (Marandu, Xaraés, Piatã and Paiaguás) on the dynamics and concentration of the larval stages of GINs in the soil and forage strata, as well as their effects on the performance of naturally infected lambs.

## Material and methods

### Ethics in animal research

This study was approved and conducted according to the guidelines of the Ethics Committee on Animal Use of the Federal University of Rio Grande do Norte, under protocol n. 055/2016.

### Location and meteorological data

The experiment was conducted in the experimental area of the Forage Research Center (5° 53'34 "S, 35° 21'50" W), which belongs to the Federal University of Rio Grande do Norte, in Brazil. This region has a dry subhumid climate [11]. During the experimental period, which lasted from March 31 to August 31, 2017 (throughout the rainy season), the total accumulated precipitation was 1,018.5 mm, and the average temperature was 25.9 ºC [12].

### Experimental area and animals

**Fig 1:**
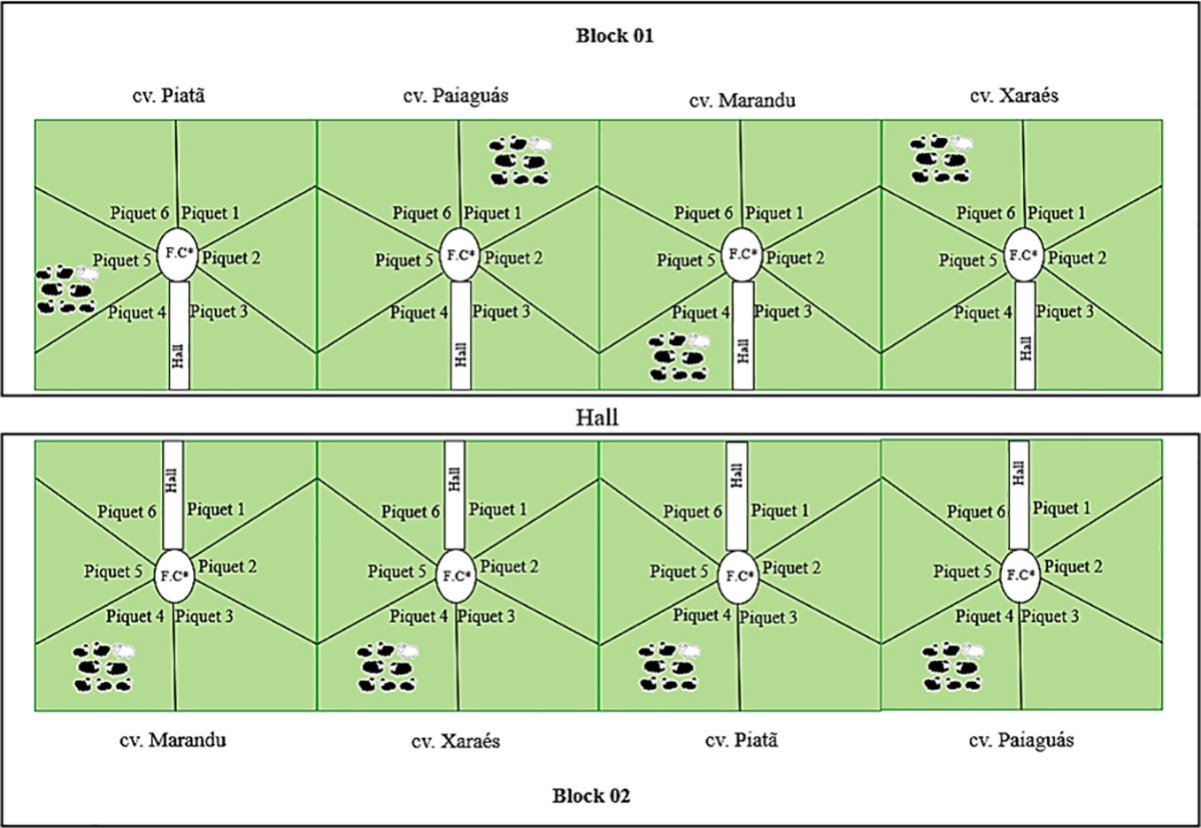
Sketch of the distribution of experimental blocks, modules and paddocks, according to the cultivars Marandu, Xaraés Piatã and Paiaguás. * Food court.

A grazing area of 2.88 ha was divided into eight modules of 0.36 ha each, which were then subdivided into six paddocks of 0.06 ha each (Fig 1). These areas were kept free of grazing for all of 2016. Before the experimental period, the pastures were cut to the target canopy heights and fertilised according to soil analyses. Four *Brachiaria brizantha* cultivars were evaluated: Marandu, Xaraés, Piatã and Paiaguás. Intermittent stocking with pre- and post-grazing canopy heights of 40 cm and 20 cm, respectively, was used. The paddocks were also supplied with salt troughs and waterers of free access.

Two grazing cycles (C1 and C2) were assessed during the experimental period; C1 lasted 118 days, while C2 lasted only 30 days due to the higher concentration of rainfall during this cycle. A grazing cycle was considered the amount of time between the beginning of two successive grazing periods in the same pasture, and a grazing period comprised the amount of time that one group of sheep remained in the same pasture.

Altogether, 48 lambs with an initial average weight of 19.04 kg ± 0.96 were studied. They were distinguished by weight so that homogeneous groups with similar body weights could be obtained between blocks and treatments. Before the experimental period, the animals were evaluated based on faecal egg counts (FECs), and those with positive results were treated with levamisole hydrochloride at 5% (7.5 mg/kg orally). Faecal egg counts were performed again after 14 d, averaging 237.12 ± 22.9. The lambs were then introduced to the pastures for the beginning of the experimental period.

Regulator ewes aged between two and four yr and weighing 45.5 kg ± 6.16 controlled the pre- and post-grazing target heights. These regulator ewes were in post-lactation, mating and pregnancy periods. Such animals were monitored for FECs (animals with FECs > 500 were drenched) and faecal cultures.

A semi-intensive production system was used, which means that the animals were allowed out to graze during the daytime (from 7 a.m. to 4 p.m.) and sheltered at night. The lambs were also kept in different stalls according to each treatment group and, beginning in the 10th week of the experiment, received a protein energy supplement at 0.5% of their average body weight (organic matter basis) for daily gains of 150 g [13]. The concentrate supplement consisted of 63.75% ground corn, 30.54% soybean meal, 2.29% feed grade urea and 3.42% mineral supplement.

The lambs were evaluated every seventh morning before they entered the paddocks. Evaluations of the environmental components (pasture and soil) were performed at each rotation of the animals among the paddocks based on the target pre- (40 cm) and post-grazing heights (20 cm).

### Parasitological examinations

Faecal samples were collected directly from the rectums of the animals every seven days, placed in individual plastic bags and identified. Furthermore, faecal egg counting was effected according to the methodology of [14], which [15] modified. Nematode genera were identified by faecal cultures after each faeces collection. Faecal samples from each experimental group of six lambs were combined and analysed according to the adopted technique [16]. Infective third-stage larvae (L3) were identified according to the morphological characteristics that [17] describe, based on the examination of 100 larvae. The results were expressed as a percentage of each genus found.

### Haematological examination and FAMACHA^©^ scoring

Blood samples were collected via jugular venipuncture and placed in 4 ml vacuum tubes that contained ethylenediaminetetraacetic acid. The mean corpuscular volume (MCV) was determined using the centrifugation of microhematocrit tubes at 1,500 rpm for 10 min [18]. The lambs were assessed with the FAMACHA^©^ method [19]. The colour of the ocular conjunctiva of all the animals was scored on a scale from 1 to 5 (red, red-pink, pink, pink-white and white, respectively) that corresponded to the levels of anaemia in the lambs.

### Weighing and body condition

The lambs were weighed on a digital scale after a solid 16 hr fast every 15 d. The body condition score (BCS) was established through palpating the thoracic and vertebral regions of the spine to measure the amount of fat and musculature at the angle formed by the transverse and spinous processes of the lumbar spine. This score was based on a scale from 1 to 5, where 1 = very thin, 2 = thin, 3 = slightly fat, 4 = fat and 5 = obese [20].

### Quantification and identification of larvae in pasture and soil samples

#### Pasture

The GIN larvae were recovered from five random samples of grass per paddock. These larvae were also collected from 5 a.m. to 7 a.m., as early morning is when they are found in the highest parts of the canopy. The samples were collected using a 0.50 m^2^ square in the pre- and post-grazing periods. In the pre-grazing period, the samples were cut from the upper canopy layer (20–40 cm) and lower canopy layer (0–20 cm), while in the post-grazing period, the samples were cut close to ground level.

These samples were placed in plastic bags, identified and processed according to the modified method of [3] to allow the larvae from the pasture to recover. All larvae found in the final sample were counted and classified into larval stages (L1 or L2) and infective larvae (L3). L3 larvae were identified by genus using their morphological characteristics [17].

#### Soil

Soil and pasture samples were collected simultaneously. Soil samples were collected with a garden shovel at a depth of 0–5 cm (under clumps) in the pre- and post-grazing periods. The recovery of the soil larvae was adapted from the method of [21]. Overall, 50 g of soil samples were placed on sieves with No. 7 mesh (148x70 mm), which were then placed on 180 mL disposable cups that contained room temperature water to allow larval migration from the soil to the water. After 24 hr, the sediment was collected with a Pasteur pipette and transferred to 15 mL test tubes for further sedimentation. The sediment was then collected for the quantification and identification of the larvae [17].

### Production, structural components and nutritional value of forage

Pasture samples were collected at each rotation of animals among paddocks to determine forage mass, pasture density, structural components and nutritional value. Six random samples from each paddock were collected with a 1 m^2^ square. The samples were weighed (green weight), and each sample was subsampled (50% of the harvested green weight), packed in a paper bag and oven-dried at 55 ºC until constant weight was obtained. Dried samples were weighed again.

Three subsamples representative of the initial samples were collected to determine the forage mass. These subsamples were divided into three fractions: leaf blade, stem (stem and sheath) and dead material. These components were then weighed and oven-dried at 55 ºC until constant weight was achieved. The results of the forage mass were converted to kg/ha of DM, and morphological components were expressed as a percentage of the forage mass. Finally, the forage samples were ground in a Wiley mill to pass a 1 mm sieve to determine the nutritional value using near-infrared reflectance spectroscopy [22].

### Statistical analysis

The experimental design used randomised blocks with 12 repetitions (animals) for variables concerning animals and 12 repetitions (paddocks) for variables concerning plants and soil, respectively. The FECs were transformed into a log using log10 (x + 1), as they are not normally distributed. Furthermore, qualitative data (L3) were transformed into a logarithm and then submitted to analysis by Pearson's chi-square test, with a degree of freedom equal to 1 and an error of 0.05, using PROC FREQ [23]. Significant interactions have been cut. The data were analysed as a factorial design to study the effect of cultivars, cycles and canopy strata; the other data were submitted to analysis of variance, followed by the F test (α = 0.05). The Tukey test was then used to assess the main and interaction effects at a significance level of 5%.

## Results

### Parasite load and animal performance

Treatments had a significant effect on the FECs. Lambs grazing Marandu grass had the highest level of infection (*P* < 0.05) compared to the other groups in C2 and between cycles. Lambs grazing Xaraés and Piatã grasses had lower parasite loads in C2 (*P* < 0.05) than C1 (Table 1).

**Table 1:** Means for fecal egg count (FEC), mean corpuscular volume (%) and body weight (kg) of lambs under intermittent stocking on different cultivars of *B*. *brizantha* for two grazing cycles.

Cultivar	FEC		MCV (%)		Body weight (kg)	
	Cycle 1	Cycle 2	Cycle 1	Cycle 2	Cycle 1	Cycle 2
Marandu	999.1 ^Ab^	1422.2 ^Aa^	24.83 ^ABb^	28.72 ^Aa^	23.19 ^Ab^	29.94 ^Aa^
Xaraés	861.5 ^Aa^	635.71 ^Ba^	22.66 ^Bb^	28.00 ^Aa^	21.79 ^Bb^	26.91 ^Ba^
Piatã	997.0 ^Aa^	817.1 ^Ba^	25.96 ^Aa^	29.00 ^Aa^	23.28 ^Ab^	29.71 ^Aa^
Paiaguás	947.4 ^Aa^	977.1 ^ABa^	24.53 ^ABa^	28.00 ^Aa^	22.45 ^ABb^	27.69 ^Ba^

Means followed by equal uppercase letters in the column and lowercase letters in the row do not differ by Tukey's Test at a significance level of 5%; CV%: FEC = 52.37; mean corpuscular volume = 47.53; body weight = 17.41.

Lambs grazing Xaraés grass had a lower MCV (*P* < 0.05) compared to others in C1. No significant differences in MCV (*P* > 0.05) were observed between groups in C2. This result demonstrates the recovery of weak lambs after the beginning of evaluations, with an MCV above 27% (non-anaemic lambs).

**Fig 2:**
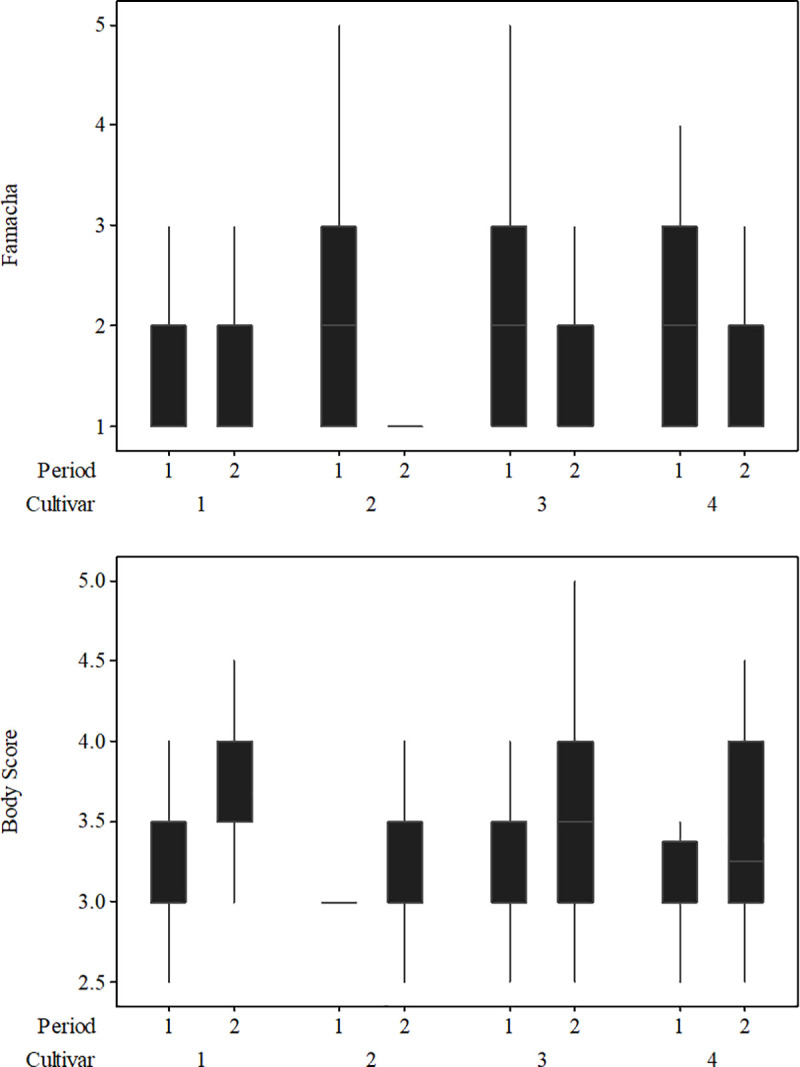
The body condition score and FAMACHA^©^ scores as a function of the cultivars (Marandu [1], Xaraés [2], Piatã [3] and Paiaguás [4]) and periods (C1 and C2, respectively).

In C1, FAMACHA^*©*^ (F) scores ranged from F1 to F5; nevertheless, most lambs had FAMACHA^©^ scores of F1, F2 and F3 (Fig 2). Lambs grazing Paiaguás grass did not exhibit F5, and 47% of them were classified as F1. In C2, the animals did not exhibit F5, and most of them were classified as F1 (67.2%), followed by F2 (25.7%) and F3 and F4 (7.1%).

A significant difference was observed in the body weight of lambs (*P* < 0.05) kept on different cultivars. The average daily gain of lambs on Xaraés grass was lower than that of lambs on Marandu, Piatã and Paiaguás grasses (Table 1), totalling 5.15% in C1 and 7.57% in C2. Treatments had a significant effect (*P* < 0.05) on grazing cycles for all groups.

Most lambs in C1 had a BCS of 3. Body condition scores of 3.5 and 4 occurred most often among lambs on Marandu grass, followed by lambs on Piatã and Paiaguás grasses. Conversely, BCSs that ranged from 2.5 to 3 occurred most often among lambs on Xaraés grass; however, these lambs maintained a BCS of 3 in C2. No BCS of 1 occurred throughout the experimental period (Fig 1). In C2, 10% of the lambs kept on Marandu and Piatã grasses had a BCS of 4.5.

The genera of GIN larvae identified in the faecal cultures of all groups and cycles include *Haemonchus*, *Trichostrongylus*, *Strongyloides* and *Oesophagostomum*. Nevertheless, *Haemonchus* spp. were the most prevalent nematode (92.01%) in the samples of all groups and cycles (Table 2), whereas *Oesophagostomum* spp. was the least prevalent (0.37%). The proportion of *Strongyloides* spp. and *Trichostrongylus* spp. in the samples were 3.06% and 4.55%, respectively.

**Table 2:** Infective larvae (%) identified in feces of lambs under intermittent stocking on different cultivars of *B*. *brizantha* for two grazing cycles (C1 and C2).

Genus	Marandu		Xaraés		Piatã		Paiaguás	
	C1	C2	C1	C2	C1	C2	C1	C2
*Haemonchus* spp.	91.5	98.0	88.6	85.9	91.4	93.3	94.6	92.8
*Trichostrongylus* spp.	6.0	0.7	6.1	1.8	4.5	1.5	3.4	0.5
*Strongyloides* spp.	2.2	0.7	5.0	12.1	3.8	5.0	1.8	5.8
*Oesophagostomum* spp.	0.3	0.6	0.3	0.2	0.3	0.2	0.2	0.9

Treatments had a significant effect (*P* < 0.05) on the forage mass, pasture density, leaf blade mass, stem mass and percentages of leaf blades and dead material (*P* < 0.05) (Table 3). The highest forage mass (2,567.95 kg/ha DM) and highest pasture density (123.23 g/cm^3^ DM) were observed in Marandu pastures. Conversely, the Paiaguás cultivar had not only the lowest forage mass and pasture density but also the highest percentage of dead material among the cultivars. The Piatã cultivar had the highest percentage of leaf blades and the lowest percentage of dead material. Pasture density was similar between Piatã (102.89 g/cm^3^ DM), Paiaguás (94.20 g/cm^3^ DM) and Xaraés grasses (133.71 g/cm^3^ DM).

**Table 3:** Forage mass, pasture density and structural characteristics of the canopy of different cultivars of *B*. *brizantha* grazed by sheep.

Variables	Cultivar				SEM	P-value
	Marandu	Xaraés	Piatã	Paiaguás		
FM (kg/ha^-1^.DM)	2567.95a	2385.22ab	2100.01bc	1951.56c	489.00	0.005
FDMD (g/cm^3^)	122.23a	113.71ab	102.89bc	94.20c	20.77	< .0001
LBM (kg/ha^-1^.DM)	990.70a	946.10a	918.50a	642.93b	273.75	< .0001
SM (kg/ha^-1^.DM)	824.16a	736.72ab	688.06ab	558.68b	289.08	0.0450
DMM (kg/ha^-1^.DM)	766.09	734.71	542.79	749.95	311.59	0.4055
Leaf blade (% of FM)	38.83ab	40.04ab	44.27a	33.52b	10.05	0.0047
Stem (% of FM)	32.28	30.49	32.24	27.90	9.78	0.6397
Dead material (% of FM)	30.27ab	30.87ab	25.84b	38.58a	11.94	0.0055
Leaf blade: stem	1.60	2.35	2.01	1.34	1.43	0.1056
Live: dead material ratio	5.79	6.66	9.87	5.41	7.21	0.5659
	---------------------------Stem (% of FM) ----------------------------					
Stratum		Marandu	Xaraés	Piatã	Paiaguás	cv.*st (P-value)
0–20 cm		37.70±13.48aA	40.85±10.89aA	41.49±12.09aA	27.96±10.37aA	0.0053
> 20cm		26.86±10.01aA	20.14±8.13bB	22.99±8.24bB	27.83±6.62aA	

FM = forage mass; FDMD = forage dry matter density; LBM = leaf blade mass; SM = stem mass; DMM = dead material mass; CV = cultivar; ST = stratum. Means followed by different lowercase letters in the row and uppercase letters in the column differ by Tukey's test at the significance level of 5%.

A significant interaction between cultivars and stratum influenced stem percentage (*P* < 0.05). Marandu and Paiaguás grasses had higher stem percentages in the stratum > 20 cm, whereas no effect occurred at 0–20 cm (*P* > 0.05). No difference between strata for Marandu and Paiaguás cultivars was observed (*P* > 0.05).

Regardless of the cultivar, no difference in the chemical composition of the leaf blade and stem, respectively, was observed for crude protein (7.75% ± 0.42; 5.34% ± 2.39), neutral detergent fibre (72.72% ± 1.27; 77.11% ± 1.02), acid detergent fibre (36.88% ± 0.77; 48.36%± 5.25), acid detergent lignin (3.06% ± 0.06; 4.43% ± 0.53), organic matter (92.37% ± 0.52; 92.41% ± 0.31) and organic matter digestibility (54.08% ± 2.09; 48.49% ± 3.78).

### Larvae on grass

Altogether, 8,181 larvae/100 g of green material were recovered from all cultivars in the two grazing cycles. L1/L2 and L3 larvae of the genera *Haemonchus* and *Trichostrongylus* were identified in all cultivars (Table 4). The highest percentages of larvae were observed in Marandu and Paiaguás grasses, whereas Piatã grass had the lowest percentage of larvae (*P* < 0.05). Moreover, the percentage of L3 larvae of both genera mentioned above were higher in Marandu and Paiaguás cultivars (*P* < 0.0001) than the other cultivars. Xaraés and Piatã grasses had the highest proportion of L1/L2 stages (*P* < 0.05).

**Table 4:** Percentage and frequency of gastrointestinal nematode larval stages found in different cultivars of *B*. *brizantha* grazed by sheep.

Cultivar	L1/L2 (%)	L3H (%)	L3T (%)	Total (%)
Marandu	23.47 (41.27)	56.74 (38.05)	48.60 (37.14)	33.25 (94.83)
Xaraés	31.02 (54.70)	4.90 (3.29)	8.43 (6.44)	21.93 (62.55)
Piatã	31.38 (55.33)	1.87 (1.26)	2.75 (2.10)	20.46 (58.34)
Paiaguás	14.20 (25.03)	36.48 (24.46)	40.22 (30.73)	24.36 (69.47)
P-value	0.0031	< .0001	< .0001	0.0105

L1/L2 = first and second free-living stages of gastrointestinal nematode larvae. L3H = *Haemonchus* sp.; L3T = *Trichostrongylus* sp. Pearson's chi-square test (significance level of 5%).

The frequency of larvae was higher in C1 (*P* < 0.0001) than C2 for all cultivars (Table 5). The percentage of larvae was lower in C2 compared to C1, and no L3 larvae of *Haemonchus* or *Trichostrongylus* were found in the Xaraés cultivar. The number of total larvae and L1/L2 frequency was similar among the cultivars in C1 and C2 (*P* > 0.05).

**Table 5:** Percentage and frequency of gastrointestinal nematode larvae found in plants of different cultivars of *B*. *brizantha* grazed by sheep according to grazing cycle.

Cultivar	Cycle 1 (%)				Cycle 2 (%)			
	L1/L2	L3H	L3T	Total	L1/L2	L3H	L3T	Total
Marandu	21.52 (37.95)	56.29 (37.75)	47.81 (36.53)	31.81 (90.73)	1.89 (3.32)	0.45 (0.30)	0.79 (0.60)	1.44 (4.10)
Xaraés	29.17 (51.43)	4.90 (3.29)	8.43 (6.43)	20.68 (58.98)	1.86 (3.27)	0 (0)	0 (0)	1.25 (3.57)
Piatã	28.83 (50.83)	1.16 (0.78)	1.11 (0.85)	18.56 (52.95)	2.55 (4.49)	0.71 (0.48)	1.64 (1.26)	1.89 (5.39)
Paiaguás	12.34 (21.75)	35.77 (23.99)	35.62 (27.21)	21.67 (61.79)	1.86 (3.28)	0.71 (0.48)	4.60 (3.51)	2.69 (7.67)
P-value	0.7606	0.0264	0.0074	0.3548	0.7606	0.0264	0.0074	0.3548
Cycle	L1/L2 (%)		L3H (%)		L3T (%)		Total (%)	
1	91.85 (161.96)		98.13 (65.79)		92.97 (71.03)		92.73 (264.45)	
2	8.15 (14.37)		1.87 (1.26)		7.03 (5.37)		7.27 (20.74)	
P-value	< .0001		< .0001		< .0001		< .0001	

L1/L2 = first and second free-living stages of gastrointestinal nematode larvae. L3H = *Haemonchus* sp.; L3T = *Trichostrongylus* sp. Pearson's chi-square test (significance level of 5%).

The Marandu and Paiaguás cultivars had the most larvae in the pre- and post-grazing periods compared to the other cultivars (Table 6). In the pre-grazing period, the lower canopy stratum had the highest concentration of larvae (*P* < 0.0001). The presence of larval stages L1/L2 in the upper stratum of the canopy was detected mainly in the Xaraés and Piatã cultivars. When the extracts were compared, the highest concentration of larvae was observed in the lower canopy strata during the pre-grazing period, followed by the lower canopy strata in the post-grazing period and the upper canopy strata in the pre-grazing period (*P* < 0.0001).

**Table 6:** Percentage and frequency of gastrointestinal nematode larvae found in plants of different cultivars of *B*. *brizantha* grazed by sheep according to forage strata and grazing periods.

Stratum/Pre or post-grazing	Cultivar			
	Marandu	Xaraés	Piatã	Paiaguás
Lower canopy stratum (< 20 cm)				
L1/L2	10.55 (18.59)	18.25 (32.18)	20.13 (35.50)	8.44 (14.88)
L3H	32.26 (21.63)	2.48 (1.66)	0.71 (0.48)	16.25 (10.89)
L3T	25.59 (19.55)	4.83 (3.69)	0 (0)	23.04 (17.61)
Total	16.52 (47.11)	12.81 (36.52)	12.48 (35.86)	13.07 (37.28)
Upper canopy stratum (> 20 cm)				
L1/L2	2.06 (3.63)	2.13 (3.75)	2.55 (4.49)	2.72 (4.79)
L3H	5.97 (4.00)	0 (0)	0.71 (0.48)	1.75 (1.18)
L3T	2.99 (2.28)	0 (0)	1.64 (1.26)	5.39 (4.11)
Total	3.27 (9.32)	1.42 (4.05)	1.89 (5.40)	3.67 (10.48)
Post-grazing (0–20 cm)				
L1/L2	10.80 (19.05)	10.65 (18.77)	8.70 (15.33)	3.04 (5.37)
L3H	18.51 (12.41)	2.42 (1.62)	0.45 (0.30)	18.48 (12.39)
L3T	20.02 (15.30)	3.60 (2.75)	1.11 (0.85)	11.79 (9.00)
Total	13.46 (38.38)	7.71 (21.98)	6.09 (17.36)	7.61 (21.71)
Stratum/Pre or post-grazing	L1/L2 (%)	L3H (%)	L3T (%)	Total (%)
Post-grazing	39.41 (37.83)	33.95 (14.03)	41.42 (18.05)	38.35 (60.36)
Lower stratum—Pre-grazing	52.90 (50.77)	56.36 (23.30)	53.34 (23.24)	53.14 (83.64)
Upper stratum—Pre-grazing	7.68 (7.38)	9.69 (4.00)	5.24 (2.28)	8.50 (13.38)
P-value	< .0001	0.0012	0.0003	< .0001

L1/L2 = first and second free-living stages of gastrointestinal nematode larvae. L3H = *Haemonchus* sp.; L3T = *Trichostrongylus* sp. Pearson's chi-square test (significance level of 5%).

### Larvae in soil

Significant differences (*P* < 0.0001) were observed for the presence of L1/L2 larvae in the soil. The highest percentage of larvae occurred in soil planted with Marandu grass compared to other substrates. Piatã and Paiaguás grasses had the least larvae in the soil (Table 7).

**Table 7:** Percentage and frequency of gastrointestinal nematode larvae found in soils planted with different cultivars of *B*. *brizantha* grazed by sheep.

Cultivar	L1/L2 (%)	L3H (%)	L3T (%)	Total (%)
Marandu	36.28 (74.47)	38.89 (10.20)	13.46 (0.78)	31.87 (128.28)
Xaraés	27.69 (56.86)	21.31 (5.59)	5.21 (0.30)	27.82 (112.00)
Piatã	24.95 (51.22)	21.62 (5.67)	53.62 (3.10)	27.06 (108.93)
Paiaguás	11.08 (22.74)	18.18 (4.77)	21.71 (1.60)	13.25 (53.34)
P-value	< .0001	0.4280	0.3726	< .0001

L1/L2 = first and second free-living stages of gastrointestinal nematode larvae. L3H = *Haemonchus* sp.; L3T = *Trichostrongylus* sp. Pearson's chi-square test (significance level of 5%).

The genera *Haemonchus* and *Trichostrongylus* were discovered in the soil. The highest percentage of larvae was found in soil planted with Marandu grass (*P* < 0.0001). Phytonematodes of the genera *Helicotylenchus* (99%) and *Criconemoides* (1%) were also found in soil samples.

The highest concentrations of larvae in the soil were observed in C1 for all cultivars (*P* < 0.0001) (Table 8). The soil planted with Marandu grass had the highest concentrations of larvae, especially L3 of *Haemonchus* spp. The lowest percentages of larvae were observed in the Paiaguás grass. No larvae of *Trichostrongylus* spp. were discovered in Xaraés or Paiaguás grasses in C1; nevertheless, this parasite was found in Piatã grass in C2.

**Table 8:** Percentage and frequency of gastrointestinal nematode larvae found in soils planted with different cultivars of *B*. *brizantha* grazed by sheep according to grazing cycle.

Cultivar	Cycle 1				Cycle 2			
	L1/L2 (%)	L3H (%)	L3T (%)	Total (%)	L1/L2 (%)	L3H (%)	L3T (%)	Total (%)
Marandu	16.75 (34.38)	30.65 (8.04)	13.46 (0.78)	20.79 (83.59)	19.53 (40.10)	8.23 (2.16)	0 (0)	11.11 (44.69)
Xaraés	14.87 (30.53)	10.78 (2.83)	0 (0)	19.21 (77.22)	12.82 (26.33)	10.53 (2.76)	5.21 (0.30)	8.65 (34.78)
Piatã	15.44 (31.69)	15.01 (3.94)	25.55 (1.48)	18.60 (74.80)	9.51 (19.53)	6.61 (1.73)	28.08 (1.62)	8.37 (33.66)
Paiaguás	6.88 (14.12)	11.57 (3.03)	0 (0)	9.71 (39.03)	4.20 (8.63)	6.61 (1.73)	27.71 (1.60)	3.56 (14.31)
Cycle	L1/L2 (%)		L3H (%)		L3T (%)		Total (%)	
1	53.93 (110.72)		68.02 (17.83)		39.01 (2.26)		68.31 (274.64)	
2	46.07 (94.58)		31.98 (8.38)		60.99 (3.53)		31.69 (127.43)	
P-value	0.2600		0.0649		0.5971		< .0001	

L1/L2 = first and second free-living stages of gastrointestinal nematode larvae. L3H = *Haemonchus* sp.; L3T = *Trichostrongylus* sp. Pearson's chi-square test (significance level of 5%).

L3 larvae of *Haemonchus* spp. were not detected in soil planted with Xaraés and Paiaguás grasses in C1 but were in C2. No L3 larvae of *Trichostrongylus* spp. were found in soil planted with Marandu grass in C2.

Similar frequencies of total GIN larvae were observed in the soil of different cultivars (*P* > 0.05) in the pre- and post-grazing periods (Table 9). However, the percentage of total larvae was highest in Marandu grass, followed by Xaraés, Piatã and Paiaguás grasses. Nevertheless, the dynamics of some GIN genera differed between cultivars and pre- and post-grazing periods. For instance, L3 larvae of the genus *Trichostrongylus* were discovered in soil planted with Marandu and Paiaguás grasses during the pre-grazing period and soil planted with Xaraés and Piatã grasses during the post-grazing period. Furthermore, L3 larvae of the genus *Haemonchus* were found in soil planted with Piatã grass only during the pre-grazing period.

**Table 9:** Percentage and frequency of gastrointestinal nematode larvae found in soils planted with different cultivars of *B*. *brizantha* grazed by sheep according to grazing periods.

Cultivar	Pre-grazing (%)				Post-grazing (%)			
	L1/L2	L3H	L3T	Total	L1/L2	L3H	L3T	Total
Marandu	17.59 (36.11)	30.65 (8.04)	13.46 (0.78)	15.97 (64.27)	18.69 (38.37)	8.23 (2.16)	0 (0)	15.90 (64)
Xaraés	15.18 (31.16)	14.23 (3.73)	0 (0)	14.65 (58.99)	12.52 (25.70)	7.08 (1.86)	5.21 (0.30)	13.17 (53)
Piatã	12.76 (26.20)	0 (0)	0 (0)	13.23 (53.26)	12.19 (25.02)	21.62a (5.67)	53.62 (3.10)	13.83 (55.68)
Paiaguás	4.69 (9.62)	12.24 (3.21)	27.71 (1.60)	6.56 (26.40)	6.39 (13.12)	5.94 (1.56)	0 (0)	6.69 (26.93)
Grazing period	L1/L2 (%)	L3H (%)	L3T (%)	Total (%)
Post-grazing	49.79 (102.22)	42.88 (11.24)	58.83 (3.40)	49.59 (199.63)
Pre-grazing	50.21 (103.08)	57.12 (14.98)	41.17 (2.38)	50.41 (202.91)
P-value	0.9517	0.4656	0.1804	0.8699

L1/L2 = first and second free-living stages of gastrointestinal nematode larvae. L3H = *Haemonchus* sp.; L3T = *Trichostrongylus* sp. Pearson's chi-square test (significance level of 5%).

## Discussion

The structural characteristics of Marandu grass, including its high pasture density and initial prostrate growth, doubtless influenced the increase in FECs in the animals grazing this forage from C1 to C2. These characteristics result in greater canopy coverage compared to the other cultivars due to the high production of green biomass and dead material [10], since a significant correlation (*P* = 0.0007) occurred between FDMD and FEC.

The litter stratum covers the base of the clumps and soil, which enables an optimal microclimate (humidity, mild temperature and shading) for L3 larvae development, survival and vertical migration. Moreover, the contamination of pastures with larvae in C1 contributed to the elevation of the environmental parasitic load in C2. The animals were therefore reinfected.

While the Paiaguás cultivar had a lower pasture density than the other cultivars, it had a higher percentage of dead material, which helped it to cover the soil. Furthermore, particular characteristics of this forage, such as its intense basal and aerial tillering and rapid regrowth [8], may have contributed to shading, since Paiaguás grass had a higher stem percentage in the upper canopy stratum than the other cultivars, except for Marandu grass. This structure is directly associated with aerial tillering, and tillers are also components of the stem mass in this stratum. Aerial and basal tillers have characteristics that can influence canopy growth and structure. Overall, aerial tillers are tender and have not only a higher leaf/stem ratio but also a better nutritional value than basal tillers [24].

The animals were kept on grazing areas for longer periods in C1 (130 days of occupation) and shorter periods in C2 (22 days of occupation), which may have also contributed to the infection level of the lambs. Parasite load in animals is directly associated with the number of larvae in the pasture.

Lambs grazing Xaraés and Piatã grasses had lower means for FECs in C2 than C1. Some factors that facilitated climate agents, such as sunlight and air circulation, may have contributed to the desiccation of larval forms of GIN [24–26] during post-grazing periods. The contamination of the pasture thus reduced in the subsequent pre-grazing period. Piatã grass clumps were more spaced, less dense and more open, with uncovered soil areas and a lower percentage of dead material compared to the other cultivars. Furthermore, the lambs consumed the entire leaf blade and left only the stems, which resulted in greater exposure of the grazing area to environmental factors.

Marandu, Xaraés and Piatã cultivars formed open clumps with a high production of leaf mass, which was the most grazed component. The pasture densities of Marandu and Xaraés cultivars were similar due to the intrinsic characteristics of these grasses, such as little branching and rapid basal tillering. However, the Xaraés cultivar grows in well‐defined clumps [8], allowing sunlight to reach the sides of the clumps, where the animals walk and deposit contaminated faeces.

The highest and lowest leaf blade/stem ratios were observed in Xaraés and Paiaguás cultivars, respectively. Leaf blade/stem ratio and the arrangement of leaves in the canopy are critical factors in feed selection for pastures [27] since animals prefer to consume the green components of plants, which are more nutritious than other components [28]. In addition, the area between the leaf sheath and stem where L3 larvae are protected reduces due to the reduced formation and retention of a film of moisture on these morphological components.

Inverse larval migration occurs when the conditions of the medium are unfavourable [29]; L3 larvae move to leaf sheaths and soil where they are partially protected from diverse environmental conditions that cause dissection. These characteristics of the Xaraés cultivar probably influenced the reduction of the mean FEC in C2. The level of pasture larval contamination affected the parasite loads of the animals. Xaraés and Piatã grasses indicated the lowest L3 contamination and lambs with the lowest contamination levels.

The high frequency of *Haemonchus* spp. and *Trichostrongylus* spp. in all cultivars and cycles corroborates the findings of the literature [30, 31]. These are the most common nematodes in several ecosystems, grazing methods and feeding and sanitary strategies. *Haemonchus contortus* survives extreme environments since it can develop mechanisms of defence and resistance. This nematode is thus of great concern to sheep farming for its severe impact on the host, which leads to death in a few weeks.

It is worth mentioning that the choice of forage cultivar neither controls nor facilitates the genera of GINs in pastures. Several dynamics that result from the interactions between climate and microclimate, plant characteristics, the quantity and genera that hosts deposit, the characteristics of each genus and species (the development of structures and physical and genetic mechanisms for survival) and management (grazing, nutritional and parasitic management) play a key role in controlling GINs [32].

This incident was evidenced in this study, whose aim was not to identify which forage cultivar enables or prevents the survival of a particular genus or species but rather to determine which of these nematodes are found in pastures and assess their dynamics. It was possible to evaluate the presence or absence of the same genus on different plant components, grazing cycles and plant strata. Moreover, the dispersion and dynamics proved to endure in the same environment over time, which renders nematode control more difficult. Evaluations based on the total number of larvae are therefore practical, efficient and essential in livestock systems.

The nutritional value of the forage associated with supplementation, especially the protein content, may have contributed to the development of resilience in the lambs. This result was observed particularly in the lambs kept on Marandu and Paiaguás grasses due to the increase in FECs in C2, which did not affect the MCV, FAMACHA^©^ score, BSC or weight gain. Changes in the MCV can be observed in the colour of the ocular conjunctiva in herds with more than 60% of the parasite load attributable to *Haemonchus* by using the FAMACHA^©^ method [33, 34, 35]. FAMACHA^©^ is thus an effective and reliable method to be applied in this study since the frequency of *Haemonchus* spp. was greater than 90%.

The concentrate supplement was first supplied to the animals in the 10th week of the experiment. The lack of this supplementation beforehand may have contributed to the identification of animals with F4 (≤ 5%) and F5 (≤ 1%). While the lambs did not initially receive concentrate supplements, the colouration of the ocular conjunctiva was adequate in most of them. Nevertheless, the animals became more resilient to infections due to supplementation, the administration of anthelmintic drugs, their adaptation to the cultivars and the development of their immune systems at the beginning of C2. The ocular conjunctiva thus became redder, and the lambs were considered healthy and non-anaemic.

During C1, the keratoconjunctivitis observed in some of the animals caused irritation and redness due to increased blood circulation in the eye region, which can lead to false diagnoses of FAMACHA^©^ scores 1 and 2. This condition undoubtedly interfered with the choices of FAMACHA^©^ scores in some evaluations of this cycle. The values of the MCV and FAMACHA^©^ scores therefore disagree with the data in the literature; however, these scores had a negative correlation (*r* = −0.39).

The performance of the lambs kept in Marandu and Paiaguás pastures was not affected despite these animals having demonstrated the highest parasite loads. These lambs also reached the recommended slaughter weight of approximately 29 kg, probably due to the nutritional value of those forage cultivars [36].

The phenotypic characteristics had significant correlations (*P* < 0.0001) with, ranging from low to moderate. The variables body weight x BCS (*r* = 0.53) and FAMACHA^©^ score x MCV (*r* = −0.39) revealed a significant correlation (*P* < 0.0001) since one reflects the other. Additionally, FAMACHA© score was negatively correlated with performance traits: FAMACHA^©^ score x body weight (*r* = −0.42) and FAMACHA^©^ score x BCS (*r* = −0.35). Conversely, FAMACHA^©^ was positively correlated with FEC (*r* = 0.29).

All these phenotypic characteristics should be used together with a selection of resistant, resilient and susceptible animals as a tool for genetic improvement, which would allow a decrease in the number of antiparasitic treatments, a reduced mortality rate and an improved parasite selection process [37, 38]. Such actions contribute to parasite control and the decision-making processes for culling in successful sheep production.

The genera detected in the three components (soil, plant and animal) have a distinct population dynamic, varying with the microclimate and nutritional conditions according to each phase of the life cycle. A correlation was made between the number of larvae in pasture and the parasite load of the hosts [31, 39]. L3 sampling from the forage plant is therefore an effective and less invasive technique to determine pasture larval contamination; nevertheless, it is not accurate for determining the genera prevalent in the herd, since abiotic factors (temperature variation, humidity and pH) and biotic factors (competition, prolificacy and anthelmintic resistance) influence the survival and frequency of different genera in both media.

Climate conditions contributed to the development and migration of larvae from the faeces to the soil and pasture, where the minimum and maximum average temperatures and humidity levels were 25.57°C vs. 26.35°C and 81.53% vs. 85.40%, respectively. Rains were observed during all experimental periods, with increased volumes of rainfall from March to July (52.6 mm; 100.2 mm; 189.8 mm; 255.5 mm and 357.8 mm, respectively), despite the reduction in August (62.5 mm). Moreover, the level of herd infection varied between months. The increases in temperature and humidity provide an ideal microclimate for the development of infectious larvae from parasites in pasture. The means for FECs increased from March to May (235.42 eggs/g of faeces, 978.65 eggs/g of faeces and 1,318.8 eggs/g of faeces, respectively) with increasing rainfall and decreasing temperatures. Conversely, the means for FECs from June (1,097.5 eggs/g of faeces) to July (622.4 eggs/g of faeces) decreased due to the emergency administration of anthelmintic drugs to avoid a decline in animal performance and maintain animal welfare.

The frequency of larvae in all pastures was higher in C1 compared to C2, almost certainly since C1 coincided with the months with the highest rainfall (April through July), with an average temperature of 25.9 ºC. It provided favourable climatic conditions for the development, survival and migration of larvae. One water film is sufficient to allow L3 larvae to move on the plant [40]. Rainfall facilitates the migration of L3 from the faeces to the pasture, and high temperatures can increase the speed of the migration of larvae to the top and along the leaf blades of the pasture [32].

While paddocks with Xaraés and Piatã cultivars started C2 slightly earlier than the other treatments and benefited from a few more days of rainfall in July, the month with the highest rainfall (357.8 mm), their frequencies of larvae were lower than the others, especially Paiaguás grass. Animals kept on Paiaguás grass spent less time in C2, which occurred in mid-August, when the rains in the region were concluding. The lower precipitation in C2 compared to C1 also reduced the number of larvae in the soil, as the soil was not wet, which made survival difficult for larvae. The first and second free-living stages of GIN larvae (L1 and L2) are relatively more vulnerable to adverse climatic conditions and desiccation than L3, which are more resistant since they retain the cuticle from the previous stage (L2) [41].

However, in addition to L3 larvae, L1 and L2 larvae were recovered from the upper canopy stratum in the pre-grazing period. Two reasons may clarify this incident: The first reason concerns the wet season, in which the impact of the raindrops on the soil and the lower canopy stratum may have directed such larvae to the upper canopy stratum [42]. The second reason is that these larvae are becoming more prevalent in all plant components, as well as at 0–5 cm above the soil surface, due to larval migration from the plant to the soil. Additional research is thus necessary to understand the free-living stages of GIN larvae.

The concentration of larvae was highest in the lower canopy stratum in the pre- and post-grazing periods. About 80% of the infective larvae were found in the first 5 cm of the canopy in ryegrass pastures [43]. In a study with two forage allowances (10% and 20%) and two grazing methods (intermittent and continuous) whose objective was to evaluate the effects of ryegrass management on the risk of parasitic reinfection, the authors concluded that larval density in ryegrass pastures increases from the top to the bottom of the forage canopy regardless of the forage allowance or grazing method [44].

Knowledge of the dynamics of the free-living stages of ruminant GINs in the pastures and soil of sheep-producing regions is epidemiologically significant and should consider biotic and abiotic conditions. These data can allow one to determine the risk of infection for animals and apply strategic tools to minimise losses.

## Conclusions

The different cultivars of *Brachiaria brizantha* influence the distribution and migration of GINs in pastures, and pasture contamination proved to reflect the parasitic load of lambs. In addition to forage, soil behaves as a means of protection and a reservoir of GINs. Animal performance was not compromised despite the highest levels of contamination and infection having been detected in Marandu and Paiaguás cultivars. Based on parasitological aspects, the Xaraés and Piatã cultivars are recommended for grass-based sheep production systems over the other *Brachiaria brizantha* cultivars since they contribute to the reduction of contamination and infection.
